# Rethinking Keratoplasty for Patients with Acanthamoeba Keratitis: Early “Low Load Keratoplasty” in Contrast to Late Optical and Therapeutic Keratoplasty

**DOI:** 10.3390/microorganisms12091801

**Published:** 2024-08-30

**Authors:** Yaser Abu Dail, Elias Flockerzi, Cristian Munteanu, Nóra Szentmáry, Berthold Seitz, Loay Daas

**Affiliations:** 1 Department of Ophthalmology, Saarland University Medical Center, Kirrberger Straße, Building 22, 66421 Homburg/Saar, Germany; elias.flockerzi@uks.eu (E.F.); cristian.munteanu@uks.eu (C.M.); berthold.seitz@uks.eu (B.S.); loay.daas@uks.eu (L.D.); 2 Dr. Rolf M. Schwiete Center for Limbal Stem Cell and Congenital Aniridia Research, Saarland University, 66421 Homburg/Saar, Germany; nora.szentmary@uks.eu

**Keywords:** low load keratoplasty, acanthamoeba keratitis, optic keratoplasty, therapeutic keratoplasty, graft survival rate

## Abstract

Background: Early therapeutic penetrating keratoplasty (TKP) for Acanthamoeba keratitis (AK) is thought to have a worse visual prognosis than the delayed optical penetrating keratoplasty (OKP) after successful conservative treatment of AK. This has led to a tendency to prolong conservative therapy and delay penetrating keratoplasty in patients with AK. This retrospective series presents the results of patients with AK that underwent early penetrating keratoplasty after reducing the corneal amoeba load through intensive conservative therapy, so-called “**low load keratoplasty**” (LLKP). Patients and methods: The medical records of our department were screened for patients with AK, confirmed by histological examination and/or PCR and/or in vivo confocal microscopy, which underwent ab LLKP and had a follow-up time of at least one year between 2009 and 2023. Demographic data, best corrected visual acuity (BCVA) and intraocular pressure at first and last visit, secondary glaucoma (SG), and recurrence and graft survival rates were assessed. Results: 28 eyes of 28 patients were included. The average time from initiation of therapy to penetrating keratoplasty (PKP) was 68 ± 113 days. The mean follow-up time after LLKP was 53 ± 42 months. BCVA (logMAR) improved from 1.9 ± 1 pre-operatively to 0.5 ± 0.6 at last visit (*p* < 0.001). A total of 14% of patients were under medical therapy for SG at the last visit, and two of them underwent glaucoma surgery. The recurrence rate was 4%. The Kaplan–Meier graft survival rate of the first graft at four years was 70%. The second graft survival rate at four years was 87.5%. Conclusion: LLKP appears to achieve a good visual prognosis with an earlier visual and psychological habilitation, as well as low recurrence and SG rates. These results should encourage us to reconsider the optimal timing of PKP in therapy-resistant AK.

## 1. Introduction

**Figure 1:**
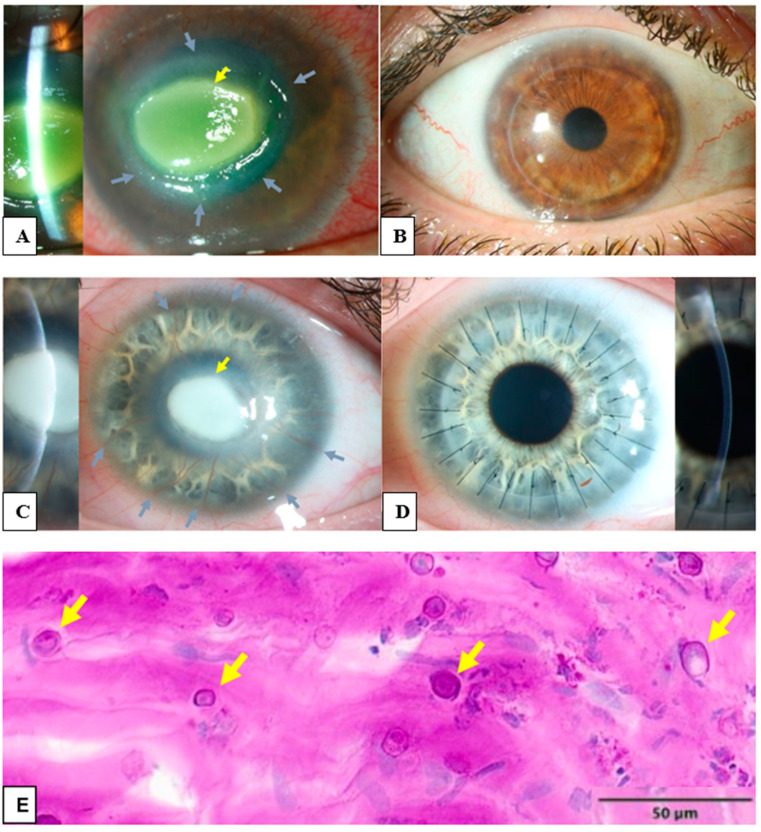
Clinical and histological findings of Acanthamoeba keratitis: (**A**) The left eye of a 45-year-old male patient with Acanthamoeba keratitis. Visual acuity by admission was hand motion. Please notice the ring infiltrate (grey arrows) and the central corneal infiltrate (yellow arrow). (**B**) Post-operative findings of the patient from (**A**). Visual acuity after ten years was 0.6 decimal. A penetrating excimer laser keratoplasty 7.5/7.6 mm was performed with corneal cryotherapy of the interface (“freezing-thawing-freezing”) before trephination. (**C**) The left eye of 21 year-old female patient with Acanthamoeba keratitis. Visual acuity by admission was hand motion. Please note the deep stromal neovascularization (grey arrows) and the central corneal infiltrate (yellow arrow). (**D**) Post-operative findings of the patient from (**C**). Visual acuity after six weeks was 0.3 decimal. A penetrating keratoplasty with mechanical trephination 7.5/7.75 mm was performed with corneal cryotherapy of the interface (“freezing-thawing-freezing”) before trephination. Please note the rapid regression of stromal neovascularization after removal of Acanthamoeba antigens with keratoplasty. (**E**) Several Acanthamoeba pathogens in the excised cornea of a patient with Acanthamoeba keratitis, some of them are marked with arrows (periodic acid-Schiff “PAS” coloring).

Acanthamoeba keratitis (AK) is a potentially sight-threatening infectious keratitis (IK) caused by Acanthamoeba species [1]. The first AK case was described by Naginton et al. after an ocular trauma [2]. AK accounts for 0–5% of all IK [3]. Incidence of AK is estimated to be 0.33–1.49 per 10,000 contact lens wearers and 0.13–2.7 per million per year in the general population [4,5,6]. While most AK occurs in contact lens (CL) wearers (71–91%) [3,7], only 5% of CL-related IK is caused by Acanthamoeba [8,9]. AK can also occur in eyes exposed to contaminated water, soil, or dust [3]. Early diagnosis of AK is decisive for a good prognosis [10,11,12]. Patients with AK may suffer from photophobia, corneal epithelial defect, perineural infiltration, ocular pain, multifocal stromal infiltration, ring-like stromal infiltration, scleritis, and ultimately loss of vision or even of the eye itself [1,13,14,15] (Figure 1).

The indication, time, and success rate of keratoplasty (KP) for treatment of AK has changed greatly over time but remains poorly defined [1]. Prior to the emergence of effective medical treatment of AK, therapeutic Keratoplasty (TKP) was mainly performed to debulk and control the infection. The prognosis of the procedure was frequently poor, with a high recurrence rate of the infection in the graft, graft failure, and loss of the eye [16,17,18,19].

In 1985, Wright et al. reported the first medical cure using a combination of topical neomycin and propamidine isethionate (Brolene^®^) [20]. Since then, medical treatments with propamidines and biguanides have revolutionized the treatment of AK with good success rates and relatively few side effects [13,21,22,23]. Approximately 75% of AK cases can be successfully treated medically with a good visual acuity and without the need for future KP [24]. Therefore, the old approach with early TKP has been abandoned in favor of a second approach with medical treatment until a cure is achieved [18]. Optical keratoplasty (OKP) for visual rehabilitation is only performed in eyes that have shown no signs of infection/inflammation for 1–3 months after medical therapy has been stopped [13,25,26]. TKP was reserved for cases with (pending) corneal perforation, persistent keratitis under prolonged medical treatment, and persistent epithelial defect. OKP had a better visual prognosis and longer graft survival than late TKP in several studies [25,26,27,28,29,30].

However, the second approach has its own drawbacks. In about 25% of patients with AK, it takes a very long time to achieve a medical cure, with several episodes of recurrence/exacerbation of infection/inflammation, and the spread of the infection/inflammation to the sclera causing severe chronic pain and possibly loss of the eye [26,31,32]. In approximately 50% of these patients, a cure may never be achieved, leading to KP being performed under very unfavorable conditions, such as in eyes with perforated corneas, major limbal stem cell deficiency (LSCD), and severe corneal neovascularization or neurotrophic keratopathy, consequently resulting in a poor visual prognosis, advanced glaucoma and, not rarely, phthisis bulbi [33,34].

On the other hand, one of our previous studies showed that an early PKP in less than five months after the onset of the symptoms in refractory cases may be associated with a better visual prognosis and survival rate than a late PKP [8]. Another study from Moon et al. also showed the possible benefits of TKP performed for treating infectious keratitis within 30 days from symptom onset compared to TKP performed after 30 days [35].

Hence, the aim of this study is to explore the effect of performing PKP after reducing the amoebic load in the cornea using intensive conservative antiamoebic therapy (AAT) that we call “**low load keratoplasty**” (LLKP), and using a larger cohort and a minimal follow-up time of one year.

## 2. Patients and Methods

**Figure 2:**
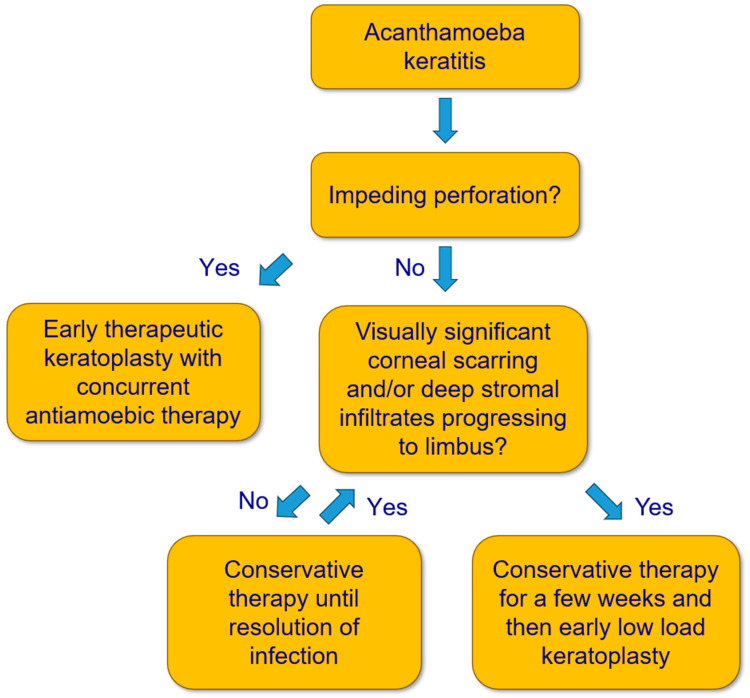
Flow chart for treatment of Acanthamoeba keratitis.

This is a retrospective case series with patients who underwent a penetrating LLKP in our department for AK. LLKP is defined as penetrating keratoplasty performed after intensive AAT for some weeks, and before discontinuation of medical therapy, in patients with deep stromal infiltrates progressing to limbus and/or visually significant corneal scarring (Figure 2). The study was performed in accordance with the Declaration of Helsinki and approved by the Ethics Committee of the Medical Association of Saarland, Germany (Nr. 84/17). Written informed consent was waived due to the retrospective nature of the study. All data were collected as part of the regular clinical examination and treatment process.

The medical records of the Department of Ophthalmology at the Saarland University Medical Center, Saarland, Germany were reviewed for patients who underwent a penetrating LLKP between 2009 and 2023 for AK, and who had a follow-up of at least one year. AK was confirmed by PCR, histology, microbiology, and/or in vivo confocal microscopy (IVCM). Excluded were patients who underwent OKP or TKP with pre-operative conservative AAT for less than one week, or who had ocular comorbidities pre-operatively, which severely reduce visual acuity (e.g., advanced glaucoma, posterior uveitis with damage to the fovea, or a history of retinal detachment with foveal involvement). In patients with two eligible eyes, only the more severely affected eye was selected. During this time period, 67 patients with AK were treated in our department (18 patients conservatively and 48 with PKP). A total of 28 eyes met the above-mentioned inclusion and exclusion criteria.

All patients were intensively treated with biguanides and diamidines pre-operatively for at least one week, ideally until the signs of inflammation decreased considerably.

Demographic data, corneal status, and time from initiation/intensification of antiamoebic therapy due to new infection/exacerbation to performing PKP (time to PKP), number, technical details of PKP, and best corrected visual acuity (BCVA) (uncorrected, contact lens, pinhole, or spectacle-corrected) were documented. Counting fingers were documented as logMAR 2, hand motion as logMAR 3, and light perception as logMAR 4 [36]. In addition, graft survival, lens status, development of glaucoma, phthisis bulbi, and enucleation were documented. As the decimal visual acuity chart is neither the standard nor easy to use for statistical analysis, BCVA was converted to logMAR and Snellen units to match the standard and to allow for comparison to the results of other studies. AK stage was defined as described by Robaei et al. [24,26,37]: Stage 1 included patients with corneal epitheliopathy only. Stage 2 included patients with corneal epithelial defect, stromal infiltration, and/or perineural infiltration. Stage 3 included patients with stage 2 and corneal ring infiltrate.

All patients were initially hospitalized and treated conservatively. Conservative therapy for AK consists of topical combination therapy of polyhexamethylene biguanide (Lavasept^®^; B. Braun, Melsungen, Germany), propamidine isethionate (Brolene^®^; Sanofi, Guildford, Surrey, UK), and neomycin sulfate 3500 IU/mL + gramicidin 0.02% + polymyxin B sulfate 7500 IU/mL (Polyspectran^®^; Alcon Pharma, Freiburg, Germany) every half-hour for 48 h, day and night, then every hour during the daytime for 3 days, and then reduced to 5–8 times a day [8].

A riboflavin-UVA cross-linking was performed in 10/28 eyes pre-operatively using the CCL 365 System (Peschke, Waldshut-Tiengen, Germany) [8].

Intraoperatively, corneal trephination of host and donor tissues was done using either the 193-nm excimer laser (MEL80; Carl Zeiss, Jena, Germany, or SCHWIND Eye-Tech-Solution, Kleinostheim, Germany) or Hessburg–Barrone vacuum trephine (Jedmed Instrument, St. Louis, MO, USA). A donor diameter with oversize of 0.1 mm for excimer laser and 0.25 or 0.5 mm for Hessburg-Barrone trephination was used [38,39,40,41]. Simultaneous cryotherapy of the host cornea was applied at the graft–host junction in 17/28 eyes before trephination (“freezing-thawing-freezing”) [8].

Post-operatively, patients were treated with Brolene 0.1%, Lavasept 0.02% (or 0.08% starting at late 2023) [23], and Polyspectran or moxifloxacine hydrochloride (Vigamox^®^) eye drops 5 times a day each. The eye drops were tapered every 6 weeks by 1x/day. Cortisone eye drops were used post-operatively twice a day until epithelial closure and then 5x/day with tapering 1x/day every 6–8 weeks [8].

Statistical analysis was performed using Microsoft Excel version 2402. Continuous data were described as mean and standard deviation. Categorical variables were described as percentages. When continuous variables were normally distributed, they were compared using the Student *t*-test. Categorical variables were compared using the Chi-Square test. A *p*-value of less than 0.05 was considered a statistically significant result. Graft survival was assessed using Kaplan–Meier analysis.

## 3. Results

**Figure 3:**
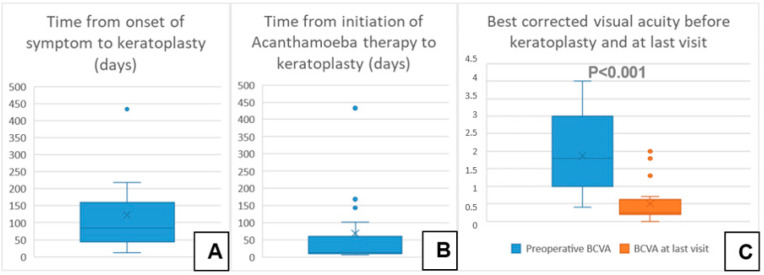
Descriptive data of the current series with a comparison of pre- and post-operative best corrected visual acuity. (**A**) Time from onset of symptom to keratoplasty in days. (**B**) Time from initiation of Acanthamoeba therapy to keratoplasty in days. (**C**) Best corrected visual acuity before keratoplasty and at last visit.

**Figure 4:**
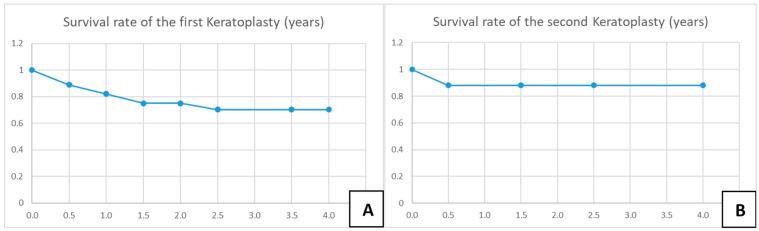
Kaplan–Meier charts for survival rate of the first and second corneal grafts. (**A**) Survival rate of the first keratoplasty in years. (**B**) Survival rate of the second keratoplasty in years.

A total of 28 eyes of 28 patients (age: 37 ± 16 years, range: 12–75 years) were eligible for analysis. A total of 46% of the eyes had AK stage 2, and 54% had AK stage 3. The mean time from onset of symptoms to LLKP was 123 ± 112 days (median: 78 days, 25th–75th percentiles: [47.0–147.0]) (Figure 3A). The average time from initiation of adequate conservative AAT to PKP was 68 ± 113 days (median: 12.5, 25th–75th percentiles: [10.0–58.0]) (Figure 3B). The mean follow-up time after LLKP was 53 ± 42 months. The mean preoperative BCVA was 1.9 ± 1 logMAR (median: 1.8, 25th–75th percentiles: [1.0–3.0]) and the post-operative BCVA at the last visit was 0.5 ± 0.6 logMAR (median: 0.25, 25th–75th percentiles: [0.2–0.48]). The difference between pre-operative and post-operative BCVA was highly significant (*p* < 0.001). BCVA results are summarized in Table 1 (Figure 3C). Of the eyes with visual acuity > 1.3, one patient had a post-operative macula edema, one developed a limbal stem cell deficiency, and one had advanced cataract. Eight eyes (29%) required a second PKP, and two of them (7%) eventually required a third PKP. Only one eye (4%) suffered a recurrence after LLKP, diagnosed through histological examination of the excised cornea. Excimer laser trephination was used in 26 eyes (93%), and Hessburg–Barrone trephination was used in 2 eyes (7%). One LLKP was performed combined with cataract surgery (4%). Round trephination was performed in 25 eyes, and elliptic trephination was performed in 3 eyes. The average diameter of the round trephinations was 8.1 ± 0.4 mm. Cryotherapy of the host cornea was performed in 17 eyes (61%). None of the eyes was enucleated. Eleven eyes required amniotic membrane transplantation after the keratoplasty to achieve epithelial stability (40%). Eight eyes developed an acute ocular hypertension (29%), four of them required topical glaucoma treatment until the last visit (14%), and two of these required one glaucoma surgical intervention each (7%). One of the repeated keratoplasties in our study was performed for recurrence of AK, one for mycotic infection, three for immunologic rejection, and three for persistent epithelial defect and melting of the transplant. The first graft survival rate at four years was 70% (Figure 4A). The second graft survival rate at four years was 87.5% (Figure 4B). Additionally, there was no significant difference between eyes which underwent cross-linking and those which did not before LLKP regarding pre- and post-operative BCVA, rates of recurrence, glaucoma, post-keratoplasty epithelial defect, and repeat keratoplasty (Table 2).

## 4. Discussion

In the following we will discuss the benefits and inherent limitations of medical therapy, the possibility of compensating for these limitations with early LLKP, how to avoid conservative and surgical overtreatment of AK patients, and the potential benefits and limitations of LLKP compared with TKP and OKP.

**Limitations of current medical treatment for AK and the early predictability of failure of medical treatment:**

The current standard treatment for AK consists of one biguanide compound (PHMB 0.02–0.08%, chlorhexidine 0.02%) as monotherapy or combined with a diamidine (propamidine 0.1% or hexamidine 0.1%) [42]. Antibiotics as eye drops are usually added as prophylaxis in cases of corneal epithelial defect and to reduce the bacterial load which provides the trophozoites with nourishment [42].

Several drugs besides biguanides and propamidines have also shown some effectiveness in treating eyes with AK. Miltefosine is an antiparasitic drug used to treat leishmaniasis, and was reported to contribute to successfully resolving several AK cases. Natamycin is an antifungal agent which also showed cysticidal effect against Acanthamoeba in vitro and was successfully used in combination with chlorhexidine to treat AK. The antifungal drug Voriconazole has also demonstrated positive results against AK [43].

Depending on the initial clinical findings of AK, the duration of medical treatment could vary widely without a guarantee of success. In general, a favorable response could be achieved within 14 days of biguanide monotherapy in approximately 80% of the cases responding to adequate conservative therapy [44]. The median conservative therapy duration for AK, including tapering time, in positively responding cases is, on average, 4.5 months compared to 11.5 months in refractory cases requiring KP [26].

The previously mentioned variation in time of treatment between responsive and refractory cases is indicative of two distinct courses of the AK (responsive vs. refractory) rather than a gradual spectrum of response to medical therapy. Early differentiation between the two courses could help guide the decision between conservative therapy alone and early surgical intervention after intensive AAT, which we called “low load keratoplasty (LLKP)”.

The main predictor of a responsive course to AAT is an early diagnosis of AK [10,11,12,45]. Another important factor is the depth of the corneal infection. Patients with deep stromal involvement have a significantly higher rate of prolonged conservative therapy, conservative treatment failure, and worse visual outcomes than those with epithelial and superficial stromal involvement only [22,46]. Interestingly, Tu et al. [46] even showed that deep stromal involvement and/or the presence of a ring infiltrate at presentation were associated with worse visual outcomes independent of symptom duration before diagnosis in 12/19 (63%) cases (odds ratio: 10.2, confidence interval: 2.9–36.1). They recommended using the presence of ring infiltrate and involvement of deep corneal stroma to predict prognosis and guide decisions for more aggressive conservative and surgical treatment, rather than the less reliable patient-reported duration of symptoms. Additionally, anterior segment ocular coherence tomography (AS-OCT) and IVCM can be very helpful in determining the depth of stromal infiltration [45,47].

There are several possible explanations for failure of treatment in AK. Although some studies have highlighted the emergence of Acanthamoeba strains resistant to propamidine [42,48,49], biguanides still show very high cytocidal activity against Acanthamoeba trophozoites and cysts in vitro, but lower activity in vivo. Therefore, resistance to therapy alone cannot explain refractory cases.

On the other hand, the high affinity of biguanides for the corneal stromal tissue appears to reduce their ability to penetrate the cornea, and consequently their bioavailability and cytocidal activity against Acanthamoeba cysts in the deep stroma [50]. This limitation of biguanides may explain the discrepancy between in vitro and in vivo sensitivity [13,48], why early diagnosis is essential for the success of conservative therapy of AK [10,11,12], and why cases with involvement of the deep stroma are unlikely to respond even to prolonged and intensive conservative therapy.

Based on the above arguments, we believe that the use of intensive conservative AAT followed by early LLKP in cases of AK involving the deep stroma would lead to a rapid and substantial reduction or elimination of the amoeba load in the superficial corneal stroma, where the drugs are most effective, and to an early surgical resection of persistent Acanthamoeba pathogens located deep in the stroma, thus utilizing the advantages of both approaches.

**Avoiding conservative and surgical overtreatment of patients with Acanthamoeba keratitis:**

An optimal approach to treating AK should take several factors into consideration. It must avoid performing LLKP early in patients who would otherwise have responded well to conservative AAT and, therefore, would not have needed keratoplasty. On the other hand, it is important to avoid delaying keratoplasty in patients with advanced AK. Otherwise, limbal and/or scleral tissues will become infected, and LSCD, deep stromal neovascularization, or secondary glaucoma may develop, with devastating consequences for the prognosis (Table 3). In addition, large-diameter corneal grafts will be unavoidable for total excision. Such grafts are associated with an increased risk of corneal rejection [39,51]. It is also important not to delay visual rehabilitation with keratoplasty unnecessarily when indicated, and to avoid prolonged conservative AAT in refractory cases, which increases the likelihood of adverse effects of such therapy [52].

Therefore, to avoid conservative and surgical overtreatment while considering the previous aspects, the following approach should be contemplated: conservative AAT remains the therapy of choice if the deep stromal corneal infiltrates do not progress to the limbus. Otherwise, LLKP should ideally be performed after a few weeks of conservative AAT, and before a large diameter graft (>8.5 mm) is required. It should also be performed in cases with visually significant corneal scarring after a few weeks of conservative therapy to achieve visual rehabilitation as soon as possible while avoiding unnecessarily long conservative AAT (Figure 2).

**Benefits and limitations of early low load keratoplasty compared to late therapeutic or optic keratoplasties:**

**Best corrected visual acuity:** BCVA varies among studies depending on the type of KP performed (TKP vs. OKP), and the indications of TKP (persistent keratitis unresponsive to AAT or corneal perforation). The BCVA achieved with LLKP appears to be better than that achieved with TKP in several published series (Table 4), especially in patients who received TKP mainly for impeding corneal perforation [26]. Interestingly, though, studies in which the TKP was performed for failed medical treatment achieved a BCVA close to the present study [25,30]. On the other hand, BCVA after LLKP appears to be slightly inferior to that after OKP. This is probably because patients in the present series who had severe complications of AK, such as LSCD, were much more likely to undergo TKP rather than OKP if KP had been actively delayed. Another reason is that several patients in the current series had their last follow-up visit right before removing the second part of corneal sutures, which is likely to lead to a better BCVA [53].

A major advantage of LLKP is that visual rehabilitation is achieved much earlier than in patients who undergo a TKP or OKP. While 75% of patients in the current study received LLKP within two months after initiating AAT, patients in whom KP is deliberately delayed received TKP after more than six months, on average, and OKP after 13–17 months, on average (Table 4). Such early rehabilitation is crucial for these young, working-age patients.

**Loss of the eye:** None of the eyes in the current series developed phthisis bulbi, glaucoma leading to reduced visual acuity, or had to undergo evisceration/enucleation within a follow-up time of 53 ± 42 months. This contrasts with the findings from the series of TKP. The enucleation rate was 4/44 (9%) in the study by Scruggs et al. [25]. In a study by Robaei et al. [26], 3–5/26 (12–19%) of patients who underwent late TKP developed phthisis bulbi eventually, and 3–5/26 (12–19%) of them developed severe glaucoma leading to reduced visual potential. This is probably because LLKP is performed on eyes with intact global integrity and without (prolonged) limbal and/or anterior chamber involvement. In addition, Acanthamoeba antigens are debulked through LLKP, as these could otherwise induce severe and recurrent episodes of keratitis, limbitis, and scleritis even when the pathogens are no longer viable [15,47,48,55].

**Recurrence of Acanthamoeba keratitis:** Recurrence rate after TKP varies widely in the literature, depending on pre-operative treatment, type of KP (DALK vs. PKP), and diagnostic criteria for recurrence. Only a positive culture for Acanthamoeba obtained from the patient’s corneal tissue can prove the presence of viable Acanthamoeba pathogens in cases of recurrence with certainty. However, the accuracy of culture is limited due to the high rate of false negatives [56]. On the other hand, positive histological findings, PCR, and IVCM results can prove the initial diagnosis with a high degree of certainty. However, they cannot prove recurrence with certainty [6,43,45,56]. The recurrence rate in the present study was 1/28 (3%), diagnosed with positive histological findings in the excised corneal button obtained during repeat PKP. The recurrence rate is estimated to be 4–41% in patients with pre-operative treatment with biguanides and with/without propamidines who underwent TKP [26,30,57]. Recurrence after OKP is estimated to be 0–22% [26,30]. Therefore, early intervention in LLKP seems to achieve a lower risk of recurrence compared to TKP and OKP. This is probably due to the early surgical removal of pathogens in the deep stroma before they spread further to the deep peripheral stroma at the limbus.

**Secondary glaucoma after keratoplasty:** After many months of unsuccessful conservative treatment, persistent wide pupils with iris base structures blocking the anterior chamber angle is not uncommon. In addition, mature cataracts do not rarely develop in long-standing AK under high dosage of conservative treatment. The rate of secondary glaucoma after KP for AK varies in the literature between 8 and 40% after TKP [30,54,57]. In the TKP series by Kitzmann et al., 1/22 patients required glaucoma surgery (5%). The rate of secondary glaucoma after OKP was 1/9 (11%) with no surgical intervention required [30]. In the current study, eight eyes developed acute ocular hypertension at some time after keratoplasty (26%), four of which required topical glaucoma treatment at the last visit (13%), and two out of these required glaucoma surgery (7%). The rate, therefore, appears to be within previously published limits.

**Graft survival rate:** Graft survival in patients with AK is known to be reduced compared to other types of infectious keratitis, and was 76% at one year, 67% at two years, and 56% at three years in a large cohort of eyes undergoing OKP or TKP [28], and 52% at two years for eyes which underwent TKP. Kashiwabuchi et al. [57] reported a graft failure rate as high as 56% after TKP. Graft survival at 12 months was 55% for the first PKP and 45% for the second PKP. Interestingly, the two-year survival rate in eyes that did not develop glaucoma after keratoplasty was approximately 70%, which was significantly better than in patients who developed glaucoma after keratoplasty [57]. Kitzmann et al. showed a survival rate of 45% at one and two years after TKP (22 patients), and a survival rate of 100% at one and two years after OKP (nine patients) [30]. Robaei et al. [26] reported a repeat PKP rate of 27% after TKP and 13% after OKP. However, it was not clear whether the eyes that underwent TKP and developed phthisis bulbi all underwent repeat keratoplasty or not. Therefore, a direct comparison with our results is not possible. In the present study, the four-year survival rate was 70%, with the majority of rejections occurring in the first year (6/8). Consequently, the rate in the current series seems to be better than that after TKP in both studies by Veugen et al. and Kashiwabuchi et al. [28,57] similar to the general rate after PKP for AK and that in eyes without glaucoma after keratoplasty, and somewhat worse than that after OKP.

Graft failure in AK appears to be multifactorial, with glaucoma [57,58], recurrent infection, immune response, and persistent epithelial defect, most likely due to neurotrophic keratitis associated with AK [56,59,60], playing a role. AK is often reported to reduce corneal sensation [56,59,60], often leading to misdiagnosis as herpetic keratitis [45]. Acanthaporin, an Acanthamoeba toxin, is cytotoxic to human nerve cells and may be one of the factors responsible for corneal nerve damage [1]. In cases of advanced AK, extensive nerve damage may explain the persistent epithelial defects after PKP, and may therefore play a role in graft failure and prognosis. The lower rate of secondary glaucoma, as well as that of recurrence after LLKP, may partly explain the lower rate of graft failure compared to TKP. In addition, LSCD and severe stromal neovascularization typically develop after long-lasting therapy-resistant disease, which results in the deterioration of graft survival.

**To wait or not to wait?**

To determine the role of the optimal timing of PKP in AK in maximizing graft survival and overall prognosis, the question is how the timing of PKP influences the development of complications associated with AK (Table 3).

The best timing should minimize the risk of all the above factors, not just some of them, while increasing the risk of others. Deliberately delaying PKP to achieve OKP may reduce graft failure due to immunologic rejection in those eyes that eventually respond to medical therapy (29–48%) [26], but it increases the risk of recurrence, glaucoma, LSCD, and perforation without avoiding surgery on the inflamed eye in those who do not respond (52–71%) [26,30].

In addition, to the best of our knowledge, there are no known modifiable factors that could sway the odds towards OKP and away from TKP in patients undergoing deliberately delayed keratoplasty with conservative AAT. Therefore, the results of OKP and TKP should not be considered as two separate groups, but as one large group resulting from deliberately delaying keratoplasty, with a subgroup that eventually responds to conservative therapy (OKP) being better than the subgroup that does not respond (TKP).

On the other hand, the current series shows that earlier LLKP may reduce secondary glaucoma and recurrence while preserving global integrity, probably resulting in an overall better prognosis and earlier visual rehabilitation than those achieved by actively delaying PKP, despite resistance to conservative therapy, and certainly a much better prognosis than in patients treated with PKP alone before the advent of effective conservative AAT.

We recommend performing LLKP in cases with deep stromal infiltrates, or central corneal infiltrates/scarring that would require future KP, ideally after a few weeks of medical treatment, as mentioned in the Methods section (Figure 2). However, we do not recommend actively postponing PKP if the clinical findings suggest otherwise, because the optimal timing of PKP should be determined based on clinical findings and should not be considered an independent prognostic factor.

The comparison between eyes which underwent cross-linking and those which did not before LLKP demonstrates that the use of cross-linking as an adjuvant therapy does not appear to increase the rate of any of the complications of AK after LLKP. On the other hand, the effectiveness of cross-linking cannot be adequately assessed based on the current data, due to the low number of eyes and to the low recurrence rate. Additionally, due to the retrospective nature of the study, a selection bias cannot be excluded.

This study is limited by its retrospective design, the lack of our own control group using other approaches, and the relatively small number of cases, which does not allow further understanding of the role of glaucoma, epithelial defect, or recurrence in the results obtained. Further studies are needed to determine the modifiable risk factors for secondary glaucoma, recurrence, persistent epithelial defects, LSCD, and immunologic graft rejection.

In summary, intensive treatment of AK with antiamoebic agents for a few weeks to reduce the amoebal load, followed by an LLKP in cases of visually significant corneal scarring or deep stromal infiltrates progressing to limbus, appears to combine two advantages: 1. The high efficacy of medical therapy in eradicating the amoeba in the superficial stroma and reducing its overall load, and 2. the efficacy of PKP in eradicating the Acanthamoeba pathogens and their antigens in the deep stroma before they spread further to the periphery or cause recurrent episodes of severe inflammation. The current approach appears to achieve good visual outcomes, with much faster visual rehabilitation, fast psychological relief, and lower recurrence and secondary glaucoma rates than TKP, similar to OKP. Although graft survival was superior to TKP, it was still inferior to OKP. Nevertheless, the overall benefits of LLKP should encourage us to reconsider the optimal timing of PKP in patients with AK therapy-resistance and to further investigate the factors behind it.

## Figures and Tables

**Table 1:** Changes in best corrected visual acuity (BCVA) pre-operatively and at last visit in patients who underwent a low load keratoplasty.

	Pre-Operative BCVA ^a^ (*n* = 28)	BCVA ^a^ at Last Visit (*n* = 28)	*p*-Value
**Mean ± SD ^b^ (logMAR)**	1.9 ± 1	0.5 ± 0.6	**<0.001 ^c^**
**Median [25th–75th percentiles]**	1.8 [1.0–3.0]	0.25 [0.2–0.48]	
**BCVA > 20/32**	0 (0%)	14 (50%)	
**BCVA 20/40–20/60**	2 (7%)	7 (25%)	
**BCVA 20/80–20/200**	7 (25%)	2 (7%)	
**BCVA < 20/200**	19 (68%)	5 (18%)	

^a^ BCVA: best corrected visual acuity. ^b^ SD: standard deviation. ^c^ *p*-value of paired Student *t*-test. **Bold**: refers to a significant *p*-value.

**Table 2:** Comparison between eyes which underwent cross-linking before low-load Keratoplasty and those which did not.

	Cross-Linking Group (*n* = 10)	No Cross-Linking Group (*n* = 18)	*p*-Value
**Pre-operative BCVA ^a^** **Mean ± SD ^b^ (logMAR)**	2.2 ± 1	1.7 ± 1	0.19 ^d^
**Post-operative BCVA ^a^** **Mean ± SD ^b^ (logMAR)**	0.5 ± 0.5	0.5 ± 0.6	1 ^d^
**Comparison of pre- and post-operative BCVA ^a^ (*p*-value)**	**0.002 ^c^**	**<0.001 ^c^**	
**Acanthamoeba keratitis stage** **(Stage 2:Stage 3)**	6:4	7:11	0.26 ^e^
**Need for AMT-Patch after Keratoplasty** **(yes:no)**	5:5	6:12	0.37 ^e^
**Acute ocular hypertension after keratoplasty** **(yes:no)**	3:7	5:13	0.9 ^e^
**Use of topical glaucoma treatment at last visit** **(yes:no)**	2:8	2:16	0.49 ^e^
**Need for glaucoma surgical intervention** **(yes:no)**	1:9	1:17	0.64 ^e^
**Recurrence rate after keratoplasty** **(yes:no)**	0:10	1:17	0.44 ^e^
**Repeat keratoplasty** **(yes:no)**	2:8	6:12	0.43 ^e^

^a^ BCVA: best corrected visual acuity. ^b^ SD: standard deviation. ^c^ *p*-value of paired Student *t*-test. ^d^ *p*-value of two-tailed Student t-test. ^e^ Chi-square test. **Bold**: refers to a significant *p*-value.

**Table 3:** Main complications of long-standing therapy-resistant Acanthamoeba keratitis.

➢ Extension of infiltration to the corneal limbus and possibly to the sclera; ➢ Excessive stromal neovascularization of the cornea; ➢ Limbal stem cell deficiency with persisting epithelial defects/ulcer; ➢ Neurotrophic keratitis; ➢ Iris atrophy with persisting pupil dilation and peripheral anterior synechiae; ➢ Mature cataract; ➢ Ciliary body detachment with ocular hypotony; ➢ Sterile chorioretinitis/retinal vasculitis.

**Table 4:** Comparison of results of best corrected visual acuity and various time spans related to keratoplasty of the current series with other studies.

		OKP ^b^	TKP ^c^	LLKP ^d^ (Current Series)
**Robaei et al.** [26]	**BCVA ^a^ > 20/32**	13 (54%)	7 (26.9%)	14 (50%)
	**BCVA 20/40–20/60**	6 (25%)	3 (11.5%)	7 (25%)
	**BCVA 20/80–20/200**	3 (12.5%)	2 (7.7%)	2 (7%)
	**BCVA < 20/200**	2 (8.3%)	14 (53.9%)	5 (18%)
	**Median time lag to keratoplasty [25th–75th percentiles] (months)**	17 [12.5–26.0]	7 [3.8–13.0]	0.4 [0.3–2.0]
**Scruggs et al.** [25]	**BCVA: median ^e^ [25th–75th percentiles] (n)**	-	0.3 [0.00–1.1] (44)	0.25 [0.2–0.48] (28)
**Kitzmann et al.** [30]	**BCVA: median ^e^ [Range] (n)**	0.1 [0–0.4] (9)	0.3 [0–2.0] (22)	0.25 [0–2.0] (28)
	**BCVA > 20/40**	8 (89%)	11 (50%)	18 (64%)
	**BCVA < 20/200**	0 (0%)	7 (32%)	5 (18%)
	**Onset of symptoms to initial keratoplasty. Mean [Range] (months)**	19 [85.0–70.0]	6 [1.0–23.0]	2.6 [0.5–5.0]
**Zhang et al.** [54] **(n = 59)**	**BCVA > 20/60**	-	29 (49%)	21 (75%)
	**BCVA 20/60–20/400**	-	18 (31%)	4 (14%)
	**BCVA < 20/400**	-	12 (20%)	3 (11%)
**Awwad et al.** [18]	**Median ^e^ [Range] (n)**	0.2 [−0.1–0.3] (12)	-	0.25 [0–2.0] (28)
	**Time from initiation of AAT to PKP. Median [Range] (months)**	13.5 [8.0–56.0]		0.4 [0.25–14.7]

^a^ BCVA: Best corrected visual acuity. ^b^ OKP: Optic keratoplasty. ^c^ TKP: Therapeutic keratoplasty. ^d^ LLKP: Low load keratoplasty. ^e^ Visual acuity is converted to logMAR for comparison.

## Data Availability

The data presented in this study are available on request from the corresponding author. The data are not publicly available due to privacy or ethical restrictions.

## References

[1] Lorenzo-Morales J., Khan N.A., Walochnik J. (2015). An update on Acanthamoeba keratitis: Diagnosis, pathogenesis and treatment. Parasite.

[2] Nagington J., Watson P.G., Playfair T.J., Mcgill J., Jones B.R., Steele A.D.M.G. (1974). Amoebic infection of the eye. Lancet.

[3] Ting D.S.J., Ho C.S., Deshmukh R., Said D.G., Dua H.S. (2021). Infectious keratitis: An update on epidemiology, causative microorganisms, risk factors, and antimicrobial resistance. Eye.

[4] Nielsen S.E., Ivarsen A., Hjortdal J. (2020). Increasing incidence of Acanthamoeba keratitis in a large tertiary ophthalmology department from year 1994 to 2018. Acta Ophthalmol..

[5] Seal D.V., Beattie T.K., Tomlinson A., Fan D., Wong E. (2003). Acanthamoeba keratitis. Br. J. Ophthalmol..

[6] Roth M., Balasiu A., Daas L., Holtmann C., Servera A., Walckling M., MacKenzie C.R., Fuchsluger T.A., Geerling G. (2023). Impact of implementation of polymerase chain reaction on diagnosis, treatment, and clinical course of Acanthamoeba keratitis. Graefes Arch. Clin. Exp. Ophthalmol..

[7] Witschel H., Sundmacher R., Seitz H.M. (1984). Amebic keratitis: Clinico-histopathologic case report. Klin. Monbl Augenheilkd..

[8] Laurik K.L., Szentmáry N., Daas L., Langenbucher A., Seitz B. (2019). Early penetrating keratoplasty à chaud may improve outcome in therapy-resistant Acanthamoeba keratitis. Adv. Ther..

[9] Meltendorf C., Duncker G. (2011). Acanthamoeba keratitis. Klin. Monbl Augenheilkd..

[10] Claerhout I., Goegebuer A., Van Den Broecke C., Kestelyn P. (2004). Delay in diagnosis and outcome of Acanthamoeba keratitis. Graefes Arch. Clin. Exp. Ophthalmol..

[11] List W., Glatz W., Riedl R., Mossboeck G., Steinwender G., Wedrich A. (2021). Evaluation of Acanthamoeba keratitis cases in a tertiary medical care centre over 21 years. Sci. Rep..

[12] Sarink M.J., Koelewijn R., Stelma F., Kortbeek T., van Lieshout L., Smit P.W., Tielens A.G.M., van Hellemond J.J. (2023). An international external quality assessment scheme to assess the diagnostic performance of polymerase chain reaction detection of Acanthamoeba keratitis. Cornea.

[13] Alkharashi M., Lindsley K., Law H.A., Sikder S. (2015). Medical interventions for acanthamoeba keratitis. Cochrane Database Syst. Rev..

[14] Szentmáry N., Daas L., Shi L., Laurik K.L., Lepper S., Milioti G., Seitz B. (2019). Acanthamoeba keratitis—Clinical signs, differential diagnosis and treatment. J. Curr. Ophthalmol..

[15] Kuennen R.A., Smith R.H., Mauger T.F., Craig E. (2010). Enucleation following treatment with intravenous pentamidine for Acanthamoeba sclerokeratitis. Clin. Ophthalmol..

[16] Hirst L.W., Green W.R., Merz W., Kaufmann C., Visvesvara G., Jensen A., Howard M. (1984). Management of Acanthamoeba keratitis: A case report and review of the literature. Ophthalmology.

[17] Hamburg A., De Jonckheere J.F. (1980). Amoebic keratitis. Ophthalmologica.

[18] Awwad S.T., Parmar D.N., Heilman M., Bowman R.W., McCulley J.P., Cavanagh H.D. (2005). Results of penetrating keratoplasty for visual rehabilitation after Acanthamoeba keratitis. Am. J. Ophthalmol..

[19] Bacon A.S., Frazer D.G., Dart J.K.G., Matheson A.S., Ficker L.A., Wright P. (1993). A review of 72 consecutive cases of Acanthamoeba keratitis, 1984–1992. Eye.

[20] Wright P., Warhurst D., Jones B.R. (1985). Acanthamoeba keratitis successfully treated medically. Br. J. Ophthalmol..

[21] Awwad S.T., Petroll W.M., McCulley J.P., Cavanagh H.D. (2007). Updates in Acanthamoeba keratitis. Eye Contact Lens..

[22] Kaufman A.R., Tu E.Y. (2022). Advances in the management of Acanthamoeba keratitis: A review of the literature and synthesized algorithmic approach. Ocul. Surf..

[23] Dart J.K.G., Papa V., Rama P., Knutsson K.A., Ahmad S., Hau S., Sanchez S., Franch A., Birattari F., Leon P. (2024). The Orphan Drug for Acanthamoeba Keratitis (ODAK) Trial: PHMB 0.08% (polihexanide) and placebo versus PHMB 0.02% and propamidine 0.1. Ophthalmology.

[24] Randag A.C., van Rooij J., van Goor A.T., Verkerk S., Wisse R.P.L., Saelens I.E.Y., Stoutenbeek R., van Dooren B.T.H., Cheng Y.Y.Y., Eggink C.A. (2019). The rising incidence of Acanthamoeba keratitis: A 7-year nationwide survey and clinical assessment of risk factors and functional outcomes. PLoS ONE.

[25] Scruggs B.A., Quist T.S., Zimmerman M.B., Salinas J.L., Greiner M.A. (2022). Risk factors, management, and outcomes of Acanthamoeba keratitis: A retrospective analysis of 110 cases. Am. J. Ophthalmol. Case Rep..

[26] Robaei D., Carnt N., Minassian D.C., Dart J.K.G. (2015). Therapeutic and optical keratoplasty in the management of Acanthamoeba keratitis: Risk factors, outcomes, and summary of the literature. Ophthalmology.

[27] Koay P.Y.P., Weng H.L., Figueiredo F.C. (2005). Opinions on risk factors and management of corneal graft rejection in the United kingdom. Cornea.

[28] Veugen J.M.J., Dunker S.L., Wolffs P.F.G., Savelkoul P.H.M., Winkens B., Biggelaar F.J.H.M.v.D., Nuijts R.M.M.A., Dickman M.M., Nctn O.B.O.T.N.C.T.N., Bartels M.C.C. (2023). Corneal transplantation for infectious keratitis: A prospective Dutch registry study. Cornea.

[29] Wu J., Xie H. (2021). Orthokeratology lens-related Acanthamoeba keratitis: Case report and analytical review. J. Int. Med. Res..

[30] Kitzmann A.S., Goins K.M., Sutphin J.E., Wagoner M.D. (2009). Keratoplasty for treatment of Acanthamoeba keratitis. Ophthalmology.

[31] Arnalich-Montiel F., Jaumandreu L., Leal M., Valladares B., Lorenzo-Morales J. (2013). Scleral and intraocular amoebic dissemination in Acanthamoeba keratitis. Cornea.

[32] Shi L., Hager T., Fries F.N., Daas L., Holbach L., Hofmann-Rummelt C., Zemova E., Seitz B. (2019). Reactive uveitis, retinal vasculitis and scleritis as ocular end-stage of Acanthamoeba keratitis: A histological study. Int. J. Ophthalmol..

[33] Stamate A.C., Tătaru C.P., Zemba M. (2018). Emergency penetrating keratoplasty in corneal perforations. Rom. J. Ophthalmol..

[34] Deshmukh R., Stevenson L., Vajpayee R. (2020). Management of corneal perforations: An update. Indian. J. Ophthalmol..

[35] Moon J., Yoon C.H., Kim M.K., Oh J.Y. (2020). The incidence and outcomes of recurrence of infection after therapeutic penetrating keratoplasty for medically-uncontrolled infectious keratitis. J. Clin. Med..

[36] Brant A., Kolomeyer N., Goldberg J.L., Haller J., Lee C.S., Lee A.Y., Lorch A.C., Miller J.W., Hyman L., Pershing S. (2023). Evaluating visual acuity in the American Academy of Ophthalmology IRIS^®^ registry. Ophthalmol. Sci..

[37] Robaei D., Carnt N., Minassian D.C., Dart J.K.G. (2014). The impact of topical corticosteroid use before diagnosis on the outcome of Acanthamoeba keratitis. Ophthalmology.

[38] Seitz B., Langenbucher A., Kus M.M., Küchle M., Naumann G.O. (1999). Nonmechanical corneal trephination with the excimer laser improves outcome after penetrating keratoplasty. Ophthalmology.

[39] Alfaro Rangel R., Szentmáry N., Lepper S., Daas L., Langenbucher A., Seitz B. (2020). 8.5/8.6-mm excimer laser-assisted penetrating keratoplasties in a tertiary corneal subspecialty referral center: Indications and outcomes in 107 eyes. Cornea.

[40] Alfaro Rangel R., Szentmáry N., Lepper S., Milioti G., Daas L., Langenbucher A., Seitz B. (2022). Large-diameter penetrating keratoplasties are mostly due to very severe infectious keratitis and cannot always prevent secondary enucleation. Klin. Monbl. Augenheilkd.

[41] Küchle M., Seitz B., Langenbucher A., Naumann G.O.H. (1999). Nonmechanical excimer laser penetrating keratoplasty for perforated or predescemetal corneal ulcers. Ophthalmology.

[42] Fanselow N., Sirajuddin N., Yin X.T., Huang A.J.W., Stuart P.M. (2021). Acanthamoeba keratitis, pathology, diagnosis and treatment. Pathogens.

[43] Shing B., Balen M., McKerrow J.H., Debnath A. (2021). Acanthamoeba keratitis: An update on amebicidal and cysticidal drug screening methodologies and potential treatment with azole drugs. Expert Rev. Anti Infect. Ther..

[44] Lim N., Goh D., Bunce C., Xing W., Fraenkel G., Poole T.R., Ficker L. (2008). Comparison of polyhexamethylene biguanide and chlorhexidine as monotherapy agents in the treatment of Acanthamoeba keratitis. Am. J. Ophthalmol..

[45] Daas L., Szentmáry N., Eppig T., Langenbucher A., Hasenfus A., Roth M., Saeger M., Nölle B., Lippmann B., Böhringer D. (2015). The German Acanthamoeba keratitis register: Initial results of a multicenter study. Ophthalmologe.

[46] Tu E.Y., Joslin C.E., Sugar J., Shoff M.E., Booton G.C. (2008). Prognostic factors affecting visual outcome in Acanthamoeba keratitis. Ophthalmology.

[47] Abu Dail Y., Daas L., Flockerzi F.A., Seitz B. (2024). Bilateral chronic contact lens-associated keratitis. Ophthalmologie.

[48] Pérez-Samonja J.J., Kilvington S., Hughes R., Tufail A., Matheson M., Dart J.K.G. (2003). Persistently culture positive acanthamoeba keratitis: In vivo resistance and in vitro sensitivity. Ophthalmology.

[49] Shi L., Muthukumar V., Stachon T., Latta L., Elhawy M.I., Gunaratnam G., Orosz E., Seitz B., Kiderlen A.F., Bischoff M. (2020). The effect of anti-amoebic agents and Ce6-PDT on Acanthamoeba castellanii trophozoites and cysts, in vitro. Transl. Vis. Sci. Technol..

[50] Vontobel S., Abad-Villar E.M., Kaufmann C., Zinkernagel A., Hauser P., Thiel M. (2015). Corneal penetration of polyhexamethylene biguanide and chlorhexidine digluconate. J. Clin. Exp. Ophthalmol..

[51] Seitz B., Langenbucher A., Naumann G.O.H. (2005). The penetrating keratoplasty. A 100-year success story. Ophthalmologe.

[52] Papa V., Van Der Meulen I., Rottey S., Sallet G., Overweel J., Asero N., Minassian D.C., Dart J.K.G. (2022). Safety and tolerability of topical polyhexamethylene biguanide: A randomised clinical trial in healthy adult volunteers. Br. J. Ophthalmol..

[53] Seitz B., Hager T., Langenbucher A., Naumann G.O.H. (2018). Reconsidering sequential double running suture removal after penetrating keratoplasty: A prospective randomized study comparing excimer laser and motor trephination. Cornea.

[54] Zhang T., Xie L., Dong Y., Cheng J. (2023). Therapeutic keratoplasty for severe Acanthamoeba keratitis: Risk factors, clinical features, and outcomes of postoperative recurrence. Graefes Arch. Clin. Exp. Ophthalmol..

[55] Lee G.A., Gray T.B., Dart J.K.G., Pavesio C.E., Ficker L.A., Larkin D.P., Matheson M.M. (2002). Acanthamoeba sclerokeratitis: Treatment with systemic immunosuppression. Ophthalmology.

[56] Dart J.K., Saw V.P., Kilvington S. (2009). Acanthamoeba keratitis: Diagnosis and treatment update 2009. Am. J. Ophthalmol..

[57] Kashiwabuchi R.T., De Freitas D., Alvarenga L.S., Vieira L., Contarini P., Sato E., Foronda A., Hofling-Lima A.L. (2008). Corneal graft survival after therapeutic keratoplasty for Acanthamoeba keratitis. Acta Ophthalmol..

[58] Reinhard T., Kallmann C., Cepin A., Godehardt E., Sundmacher R. (1997). The influence of glaucoma history on graft survival after penetrating keratoplasty. Graefes Arch. Clin. Exp. Ophthalmol..

[59] Perry H.D., Donnenfeld E.D., Foulks G.N., Moadel K., Kanellopoulos A.J. (1995). Decreased corneal sensation as an initial feature of Acanthamoeba keratitis. Ophthalmology.

[60] Illingworth C.D., Cook S.D. (1998). Acanthamoeba keratitis. Surv. Ophthalmol..

